# Prevalence and clinical characteristics of secondary hypertension in young hypertensive tertiary care patients

**DOI:** 10.1038/s41371-026-01133-w

**Published:** 2026-03-20

**Authors:** Jasmin Vesamo, Teemu J. Niiranen, Karri Suvila

**Affiliations:** 1 Department of Internal Medicine, University of Turku, Turku, Finland; 2 Division of Medicine, Turku University Hospital, Turku, Finland

**Keywords:** Risk factors, Hypertension

## Abstract

Current European guidelines recommend screening <30-year-old hypertensive patients for secondary hypertension, but the evidence behind this recommendation is limited. Our objective was to assess secondary hypertension prevalence and etiology among young adults and to determine the characteristics linked with secondary hypertension in these patients. We retrospectively studied 243 Finnish hypertensive adults aged 16–30 years (mean age 25.5 years; 49% women) evaluated at a tertiary care hospital in Finland between 2002 and 2023. Data were collected from electronic health records. Patients were classified under three hypertension subtypes: primary, secondary, or exogenic hypertension. We examined the association between participants characteristics and hypertension subtype (primary versus secondary) using logistic regression. A total of 133 patients had primary hypertension, while 98 patients had secondary hypertension. The most common causes of secondary hypertension were renal disease (n = 77) and sleep apnea (n = 13), whereas other causes were limited to 1–2 cases. Individuals with diabetes mellitus had odds of 2.79 (95% confidence interval [95% CI], 1.21-6.43; P = 0.02) for having secondary versus primary hypertension. A plasma creatinine increase of 1 mmol/l was associated with 1.03-fold (95% CI 1.01–1.04; P = 0.002) odds of secondary hypertension. Apart from renal disease and sleep apnea, other forms of secondary hypertension are extremely rare in young adults with hypertension. In this population, renal parenchymal disease and diabetes mellitus emerged as the most important risk factors for secondary hypertension. Extensive universal screening for secondary hypertension without suspicion of such condition for all hypertensive patients <30 years may be unnecessary.

## Introduction

The majority of individuals with high blood pressure (BP) have primary hypertension, in which there is no single underlying cause for elevated BP is found [1, 2]. However, some patients are diagnosed with secondary hypertension, in which a specific cause for high BP can be identified. Prior studies have reported considerably varying prevalences for secondary hypertension, ranging from approximately 1% to 16% of all hypertension cases [3–9]. The most common causes of secondary hypertension include primary hyperaldosteronism, renal artery stenosis, renal parenchymal disease, and obstructive sleep apnea. Given the potential targeted medical therapies for several causes of secondary hypertension, it is beneficial to detect these individuals as early as possible.

The previous publications on the prevalence of secondary hypertension have considerable differences in their study settings and populations, which may have resulted in mixed prevalence rates. The prevalence of secondary hypertension varies between studies from 2 to 27% among adolescents and young adults aged 12 to 30 years [7–9]. Secondary hypertension, particularly due to renal disease, appears to be somewhat more frequent in early childhood than among young adults [10, 11]. Most common etiologies for secondary hypertension among children include renal (e.g., hydronephrosis, nephrotic syndrome, glomerulonephritis, nephropathy, renal dysplasia, and renal artery stenosis) and respiratory (e.g., bronchopulmonary dysplasia and chronic lung disease) causes [11]. In the age group of 12–30 years, the most common causes of secondary hypertension include primary aldosteronism, renal parenchymal disease, renal artery stenosis due to fibromuscular dysplasia and various monogenic disorders [8, 9]. In later life, the prevalence of secondary hypertension appears to increase, most likely due to the coexistence of multiple etiological factors [5]. Most common causes for secondary hypertension in the elderly include renal artery stenosis due to atherosclerosis, renal parenchymal disease, and hypothyroidism [12, 13].

Even though hypertension guidelines emphasize the importance of distinguishing between primary and secondary hypertension particularly in younger hypertensive individuals, there is a lack of evidence on which patients should be screened for having secondary hypertension. The European Society of Hypertension (ESH) guidelines recommend screening hypertensive individuals in whom diagnostic suspicion of the condition arises for the clinician. ESH also suggest referring patients to specialized hypertension center if they are under 40 years old and have an initial BP over 160/100 mmHg in office BP measurements. [14] The European Society of Cardiology (ESC) guidelines recommend comprehensive screening of secondary hypertension among all normal weight patients with hypertension before the age of 40 years. Screening of secondary hypertension among obese patients should begin with evaluation for obstructive sleep apnea. [15] Similarly, the American guidelines recommend secondary hypertension screening in individuals with hypertension onset before 30 years of age [16]. However, there is no consensus on which secondary causes should be ruled out and which diagnostic tests should be performed for these patients.

As the evidence on the screening for secondary hypertension in young adults is limited, the objective of our study was to examine the prevalence and etiology of secondary hypertension in young Finnish adults aged 16–30 years. We also aimed to study whether certain clinical characteristics are associated with secondary hypertension among these individuals. This retrospective study analyzed 243 adult patients under 30 years old who underwent initial evaluation for hypertension at a Finnish tertiary care hospital between 2002 and 2023. We hypothesized that these individuals would have a relatively high secondary hypertension prevalence, and that certain clinical characteristics would be associated with having secondary hypertension.

## Methods

### Study population

**Fig. 1:**
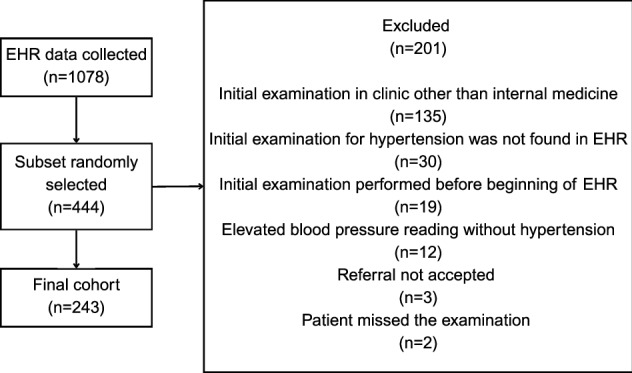
Flowchart of study sample exclusions. EHR, electronic health record.

The study participants included Finnish patients whose initial hypertension evaluation was performed at the Internal Medicine Clinic of the Turku University Hospital between January 2002 and June 2023. We included individuals aged from 16 to 30 years who were diagnosed with primary or secondary hypertension based on the 10^th^ revision of the International Classification of Diseases (ICD-10) codes. The initial cohort included 1078 participants studied retrospectively from electronic health record (EHR). From the initial cohort, we randomly selected a subset of 444 participants whose EHR data was manually reviewed. We excluded participants who did not meet the inclusion criteria (N = 201), resulting in a final sample of 243 individuals (Fig. 1). EHR data were handled confidentially using anonymized or pseudonymized information only by qualified staff necessary for data collection and analysis. The study protocol was reviewed and approved by the Wellbeing Services County of Southwest Finland’s research services (research permit code: T777/2023). Informed consent was not required due to the retrospective study setting which did not affect the care of the patients included in this study.

### Data collection

Information of participants’ previous diagnoses and current medication was either self-reported or collected from the EHR. Participants self-reported their current smoking status and number of parents with hypertension. Participants height and weight was either self-reported or measured at the office. Overweight was classified as body mass index (BMI) 25–30 kg/m^2^ and obesity as BMI > 30 kg/m^2^. We recorded the highest reported office BP measurement and the mean of home BP measurement. Mean daytime and night-time ambulatory systolic and diastolic blood pressures were recorded in participants who underwent 24-hour ambulatory BP monitoring. Nocturnal dipping was calculated as (daytime ambulatory BP – nighttime ambulatory BP/daytime ambulatory BP x 100). If available, the participants’ results on laboratory results, a standard 12-lead electrocardiogram recording, a renal artery doppler ultrasound, an echocardiogram and a home respiratory polygraphy were recorded. Suppressed plasma renin and elevated serum aldosterone were recorded as being under, within, or over the given normal range at the time of the measurement. We considered cortisol being elevated if dexamethasone suppression test, midnight salivary cortisol, or 24-hour cortisol measured from urine exceeded the normal range. Urine dipstick tests were considered positive when the result was 2+ or more. We used Sokolov-Lyon criteria to determine electrocardiographic left ventricular hypertrophy (LVH) and calculated apnea-hypopnea index (AHI) from the home respiratory polygraphy data. Renal artery stenosis was measured with ultrasound and recorded as present/absent.

### Hypertension type

Based on the findings, we classified the patients under three hypertension subtypes: primary hypertension, secondary hypertension, or exogenic hypertension. The primary hypertension subgroup included all the participants with ICD-10 code I10 with no other cause for hypertension. We included participants with gestational hypertension in the primary hypertension subgroup. Individuals with the following diagnoses were classified as having secondary hypertension: hypertension caused by renal disease (Table 1), sleep apnea, Cushing syndrome, parathyroid dysfunction, aortic coarctation, renovascular hypertension, cardiac arrhythmia, juvenile rheumatoid polyarthritis, acromegaly, replacement of thoracic aorta due to traumatic injury, and congenital malformation of the aortic valve. Participants with drug or glycyrrhizin induced hypertension were assigned the exogenic hypertension subgroup. Participants who could be classified into exogenic and secondary hypertension subgroups were assigned to the secondary hypertension subgroup. We further divided individuals in the secondary hypertension group into subgroups by the three most common causes: hypertension caused by renal disease, sleep apnea or other causes.

**Table 1:** Diagnoses among patients with secondary hypertension caused by renal disease (N = 77).

Renal disease	N
Diabetic nephropathy^a^	22
IgA nephropathy	11
Polycystic kidney disease	9
Systemic lupus erythematosus	6
Chronic kidney disease of unknown etiology	6
Unknown albuminuria	3
Nephrotic syndrome of unknown cause	2
Sarcoidosis	2
Kidney transplant	2
Unilateral nephrectomy	2
Other tubulointerstitial kidney disease	1
Tubulointerstitial nephropathy	1
Cyst of kidney, acquired	1
Goodpasture syndrome	1
Kidney stone	1
Ureteral obstruction	1
Kidney cancer	1
Chemotherapy-related kidney disease	1
Medullary cystic kidney disease	1
Renal agenesis	1
Drug-induced renal disorder	1
Granulomatous polyangiitis	1

^a^Patients were classified as having diabetic nephropathy group if they had albuminuria or decreased eGFR with diabetes. Other diagnoses were based on ICD-10 codes.

### Statistical analyses

We assessed the sample characteristics in the whole sample, in subgroups by hypertension type, and in subgroups by causes of secondary hypertension. We compared characteristics between subgroups using the X^2^ test for categorial variables and the two-sample t-test for the continuous variables. We examined the association between participants characteristics and hypertension subtype (primary versus secondary) using multivariable logistic regression. The following variables were included in the model as independent variables as they were available for most (N = 171) patients: sex, age, diabetes mellitus (DM), hyperlipidemia, hypothyroidism, asthma, BMI, office systolic blood pressure (SBP), office diastolic blood pressure (DBP), plasma potassium, plasma sodium and plasma creatine. Coronary artery disease and heart failure were not included in the model as no participants had a positive history of these diseases. We carried out all analyses using SPSS software version 29 (IBM; SPSS Statistics). A two-tailed p < 0.05 was considered statistically significant.

## Results

The characteristics of the whole study sample (mean age 25.5 years; 49% women) and in subgroups by hypertension type are presented in Table 2. A total of 133 patients (54.7%) were diagnosed with primary hypertension, while 98 patients (40.3%) were diagnosed with secondary hypertension. Sex, age, current smoking status, number of parents with hypertension, anthropometrics and BP characteristics did not differ between the subgroups. Individuals with secondary hypertension had a higher prevalence of DM, chronic kidney disease and sleep apnea than patients with primary hypertension. The most significant differences between the secondary and primary hypertension subgroups were observed for kidney function. The secondary hypertension subgroup had higher plasma creatine (p < 0.001), more dipstick hematuria (p < 0.001) and more dipstick albuminuria (p < 0.001).

**Table 2:** Characteristics of the whole study sample, and in groups by hypertension subtype.

			Hypertension type			P for primary vs. secondary
Characteristic	N with data	All	Primary	Secondary	Exogenic	
N, (%)		243	133 (54.7)	98 (40.3)	12 (4.9)	
Women, (%)	243	119 (49)	65 (48.9)	47 (48.0)	7 (58.3)	0.89
Age, (SD)	243	25.5 (3.68)	25.6 (3.66)	25.3 (3.79)	26.2 (3.07)	0.57
Medical history						
Current smoker, (%)	192	73 (38)	37 (35.2)	31 (41.3)	5 (41.7)	0.41
No. of parents with hypertension, (SD)	116	0.93 (0.64)	0.94 (0.64)	1.00 (0.62)	0.63 (0.74)	0.70
Use of antihypertensive medication, (%)	243	115 (47.3)	56 (42.1)	55 (56.1)	4 (33.3)	0.04
Number of blood pressure medications, (SD)	243	0.69 (0.91)	0.56 (0.78)	0.90 (1.06)	0.42 (0.67)	0.009
Diabetes mellitus, (%)	243	47 (19.3)	20 (15)	27 (27.6)	0 (0)	0.02
Hyperlipidemia, (%)	243	9 (3.7)	5 (3.8)	4 (4.1)	0 (0)	0.90
Coronary artery disease, (%)	243	0 (0)	0 (0)	0 (0)	0 (0)	-
Cardiac insufficiency, (%)	243	0 (0)	0 (0)	0 (0)	0 (0)	-
Sleep apnea, (%)	243	4 (1.6)	0 (0)	4 (4.1)	0 (0)	0.008
Hypothyroidism, (%)	243	9 (3.7)	8 (6)	1 (1.0)	0 (0)	0.04
Kidney insufficiency, (%)	243	37 (15.2)	2 (1.5)	35 (35.7)	0 (0)	<0.001
Asthma, (%)	243	22 (9.1)	11 (8.3)	9 (9.2)	2 (16.7)	0.81
Anthropometrics						
Body mass index, kg/m^2^ (SD)	200	30.8 (8.91)	32.3 (9.36)	29.4 (8.34)	28.0 (7.03)	0.02
Overweight, (%)	200	57 (28.5)	25 (24.8)	28 (31.8)	4 (36.4)	0.28
Obese, (%)	200	86 (43)	52 (51.5)	31 (35.2)	3 (27.3)	0.03
Blood pressure						
Office SBP, mm Hg (SD)	223	153 (20.5)	155 (17.9)	152 (23.9)	156 (18.1)	0.39
Office DBP, mm Hg (SD)	223	97.6 (14.1)	98.4 (13.9)	95.9 (14.5)	102 (12.3)	0.21
Home SBP, mm Hg (SD)	130	143 (14.8)	144 (13.2)	143 (17.1)	147 (15.8)	0.69
Home DBP, mm Hg (SD)	129	90.1 (12.2)	91.1 (11.8)	87.6 (13.3)	94.1 (8.10)	0.14
Ambulatory SBP day, mm Hg (SD)	55	149 (14.0)	149 (14.9)	151 (12.4)	156 (2.08)	0.68
Ambulatory DBP day, mm Hg (SD)	55	94.1 (10.6)	92.7 (10.6)	96.9 (10.7)	102 (6.51)	0.23
Ambulatory SBP night, mm Hg (SD)	54	130 (15.6)	129.9 (17.0)	133 (11.2)	130 (13.6)	0.59
Ambulatory DBP night, mm Hg (SD)	54	76.7 (12.0)	75.2 (12.0)	81.6 (11.7)	78.0 (11.3)	0.11
Nocturnal dipping %, (SD)	54	12.9 (6.80)	12.9 (7.17)	11.9 (5.19)	16.7 (8.56)	0.66
Biochemistry						
Serum ionized calcium, mmol/l (SD)	147	1.23 (0.057)	1.23 (0.04)	1.24 (0.06)	1.23 (0.07)	0.32
Plasma potassium, mmol/l (SD)	235	3.92 (0.39)	3.91 (0.33)	3.98 (0.46)	3.71 (0.32)	0.22
Plasma sodium, mmol/l (SD)	232	140 (2.17)	140 (2.15)	141 (2.19)	140 (2.37)	0.33
Plasma creatinine, umol/l (SD)	236	84.6 (39.2)	74.7 (22.8)	99.5 (51.6)	67.8 (13.6)	<0.001
Plasma fasting glucose, mmol/l (SD)	155	6.04 (2.37)	5.79 (1.79)	6.41 (2.96)	5.34 (0.38)	0.13
Hemoglobin A1C, mmol/mol (SD)	128	49.2 (24.6)	43.9 (17.1)	57.8 (30.6)	33.4 (2.32)	0.004
Plasma LDL-cholesterol, mmol/l (SD)	174	2.98 (2.35)	3.08 (2.87)	2.83 (1.40)	2.83 (1.07)	0.50
Suppressed plasma renin, (%)	113	29 (25.7)	23 (29.9)	2 (7.7)	4 (40.0)	0.02
Elevated serum aldosterone, (%)	111	17 (15.3)	7 (9.2)	7 (28.0)	3 (30.0)	0.03
Suppressed renin and elevated aldosterone, (%)	109	0 (0)	0 (0)	0 (0)	0 (0)	-
Elevated cortisol, %	50	7 (14)	4 (12.5)	3 (23.1)	0 (0)	0.39
Plasma thyrotropin, mU/l (SD)	186	2.23 (1.23)	2.23 (1.33)	2.19 (1.03)	2.51 (1.38)	0.81
Plasma free T4, pmol/l (SD)	142	15.3 (2.64)	15.6 (2.65)	15.1 (2.73)	14.4 (1.97)	0.29
Elevated metanephrine, (%)	93	2 (2.2)	0 (0)	1 (5.0)	1 (10.0)	0.09
Elevated normetanephrine, (%)	93	0 (0)	0 (0)	0 (0)	0 (0)	-
Urine albumin-creatinin ratio, mg/mmol (SD)	84	21.9 (79.7)	0.82 (1.08)	43.0 (109)	1.12 (1.00)	0.02
Dipstick hematuria, (%)	172	19 (11)	0 (0)	18 (23.4)	1 (11.1)	<0.001
Dipstick albuminuria, (%)	172	23 (13.4)	0 (0)	23 (29.9)	0 (0)	<0.001
Physiology and imaging						
EKG LVH, (%)	180	8 (4.4)	8 (7.8)	0 (0)	0 (0)	0.004
Renal artery stenosis, (%)	97	0 (0)	0 (0)	0 (0)	0 (0)	-
Septal wall thickness, mm (SD)	54	8.92 (1.88)	8.83 (1.43)	9.19 (2.61)	8.17 (1.26)	0.59
Apnea-hypopnea index, (SD)	15	34.7 (44.4)	1.86 (1.57)	56.8 (45.6)	0.70 (-)	0.007

*SBP* systolic blood pressure, *DBP* diastolic blood pressure, *LDL* low density lipoprotein, *EKG LVH* electrocardiographic left ventricular hypertrophy.

**Fig. 2:**
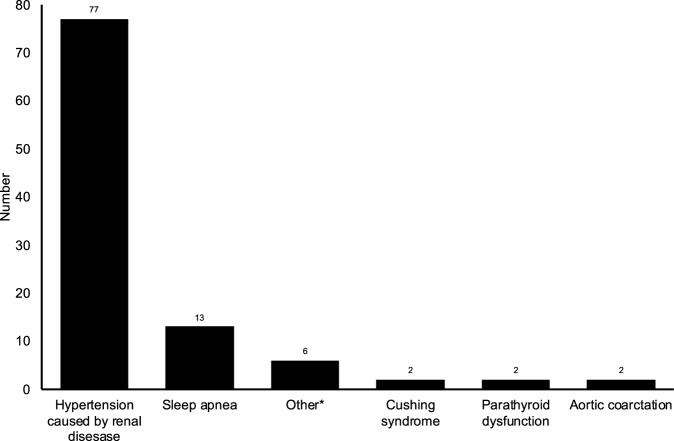
Causes of secondary hypertension. *The other causes include single cases of renovascular hypertension, cardiac arrhythmia, juvenile rheumatoid polyarthritis, acromegaly, replacement of thoracic aorta due to traumatic injury and a congenital malformation of the aortic valve.

The identified causes of secondary hypertension are presented in Fig. 2. Hypertension caused by renal disease (n = 77) was the most common cause for secondary hypertension. Other causes of secondary hypertension were markedly less common, with sleep apnea being the second most common cause (n = 13). The characteristics in subgroups by secondary hypertension causes are presented in Table 3. In all four subgroups, most participants were classified being as either overweight or obese. The prevalence of DM was high in the following subgroups: hypertension caused by other renal disease (29.9%) and sleep apnea (30.8%). The diagnoses which are included in secondary hypertension caused by renal disease are reported in Table 1. The most common renal disease diagnosis was diabetic nephropathy (n = 22), followed by IgA nephropathy (n = 11).

**Table 3:** Characteristics in subgroups by secondary hypertension causes.

	Hypertension caused by renal disease		Sleep apnea		Other secondary hypertension	
Characteristic	N with data		N with data		N with data	
N		77		13		11
Women, (%)	77	41 (53.2)	13	2 (15.4)	11	4 (36.4)
Age, (SD)	77	25.4 (3.86)	13	24.9 (3.66)	11	24.9 (3.91)
Medical history						
Current smoker, (%)	58	21 (36.2)	12	7 (58.3)	7	4 (57.1)
No. of parents with hypertension, (SD)	12	1.17 (0.39)	7	0.71 (0.76)	3	1.00 (1.00)
Use of antihypertensive medication, (%)	77	40 (51.9)	13	9 (69.2)	11	8 (72.7)
Number of blood pressure medications, (SD)	77	0.82 (1.05)	13	1.31 (1.25)	11	1.36 (1.29)
Diabetes mellitus, (%)	77	23 (29.9)	13	4 (30.8)	11	1 (9.1)
Hyperlipidemia, (%)	77	4 (5.2)	13	0 (0)	11	0 (0)
Coronary artery disease, (%)	77	0 (0)	13	0 (0)	11	0 (0)
Cardiac insufficiency, (%)	77	0 (0)	13	0 (0)	11	0 (0)
Sleep apnea, (%)	77	0 (0)	13	4 (30.8)	11	0 (0)
Hypothyroidism, (%)	77	1 (1.3)	13	0 (0)	11	0 (0)
Kidney insufficiency, (%)	77	35 (45.5)	13	0 (0)	11	1 (9.1)
Asthma, (%)	77	6 (7.8)	13	2 (15.4)	11	2 (18.2)
Anthropometrics						
Body mass index, kg/m^2^ (SD)	70	27.4 (5.91)	13	41.8 (9.63)	7	27.9 (9.27)
Overweight, (%)	70	26 (37.1)	13	1 (7.7)	7	2 (28.6)
Obese, (%)	70	18 (25.7)	13	12 (92.3)	7	2 (28.6)
Blood pressure						
Office SBP, mm Hg (SD)	70	151 (23.7)	13	155 (26.5)	10	162 (20.9)
Office DBP, mm Hg (SD)	70	95.7 (13.5)	13	96.8 (15.5)	10	98.8 (20.9)
Home SBP, mm Hg (SD)	32	141 (16.5)	9	146 (17.7)	7	150 (17.9)
Home DBP, mm Hg (SD)	31	86.9 (12.0)	9	92.5 (10.4)	7	86.6 (20.2)
Ambulatory SBP day, mm Hg (SD)	9	151 (14.4)	3	150 (2.89)	0	-
Ambulatory DBP day, mm Hg (SD)	9	98.1 (11.8)	3	93.3 (6.51)	0	-
Ambulatory SBP night, mm Hg (SD)	9	133 (12.1)	3	131 (10.1)	0	-
Ambulatory DBP night, mm Hg (SD)	9	80.9 (13.1)	3	83.7 (7.51)	0	-
Nocturnal dipping %, (SD)	9	11.8 (5.53)	3	12.3 (5.02)	0	-
Biochemistry						
Serum ionized calcium, mmol/l (SD)	63	1.23 (0.05)	5	1.25 (0.03)	8	1.27 (0.13)
Plasma potassium, mmol/l (SD)	74	4.00 (0.42)	13	3.99 (0.51)	11	3.72 (0.62)
sodium, mmol/l (SD)	74	141 (2.26)	13	141 (1.64)	11	141 (2.57)
Plasma creatinine, umol/l (SD)	76	106 (56.5)	13	81.1 (10.5)	11	80.2 (23.2)
Plasma fasting glucose, mmol/l (SD)	56	6.53 (3.22)	8	6.60 (1.32)	7	5.33 (1.18)
Hemoglobin A1C, mmol/mol (SD)	42	63.4 (32.7)	11	41.5 (8.23)	4	35.0 (7.62)
Plasma LDL-cholesterol, mmol/l (SD)	49	2.83 (1.51)	12	2.90 (1.11)	5	2.88 (0.97)
Supressed plasma renin, (%)	15	0 (0)	6	2 (33.3)	5	0 (0)
Elevated serum aldosterone, (%)	14	4 (28.6)	6	1 (16.7)	5	2 (40.0)
Supressed renin and elevated aldosterone, (%)	14	0 (0)	6	0 (0)	5	0 (0)
Elevated cortisol, %	6	0 (0)	4	1 (25)	4	3 (75.0)
Plasma thyrotropin, mU/l (SD)	53	2.24 (1.00)	11	2.21 (1.21)	5	1.42 (0.56)
Plasma free T4, pmol/l (SD)	32	15.3 (2.59)	9	15.2 (3.35)	7	14.2 (3.06)
Elevated metanephrine, (%)	12	0 (0)	4	0 (0)	4	1 (25.0)
Elevated normetanephrine, (%)	12	0 (0)	4	0 (0)	4	0 (0)
Urine albumin-creatinin ratio, mg/mmol (SD)	35	51.0 (118)	5	3.06 (4.07)	3	3.13 (2.31
Dipstick hematuria, (%)	65	17 (26.2)	8	1 (12.5)	7	0 (0)
Dipstick albuminuria, (%)	65	23 (35.4)	8	0 (0)	7	0 (0)
Physiology and imaging						
EKG LVH, (%)	50	0 (0)	13	0 (0)	8	0 (0)
Renal artery stenosis, (%)	13	0 (0)	4	0 (0)	5	0 (0)
Septal wall thickness, mm (SD)	12	8.42 (1.61)	2	14.5 (2.12)	4	8.88 (2.50)
Apnea-hypopnea index, (SD)	0	-	9	56.8 (45.6)	1	70.7 (-)

SBP, systolic blood pressure, DBP, diastolic blood pressure, LDL, low density lipoprotein, EKG LVH, electrocardiographic left ventricular hypertrophy.

Table 4 shows the multivariable odds of secondary versus primary hypertension according to different clinical characteristics. Individuals with DM had 2.79-fold (95% confidence interval [95% CI], 1.21-6.43) odds for secondary hypertension. A plasma creatinine increase of 1 mmol/l was associated with 1.03-fold (95% CI 1.01-1.04) odds of secondary hypertension. The other characteristics were not related to odds of secondary hypertension.

**Table 4:** Odds of secondary hypertension according to hypertensive tertiary care clinical characteristics.

	OR (95% CI)	P-Value
Women	1.69 (0.80-3.58)	0.17
Age, year	1.01 (0.91-1.11)	0.88
Diabetes	2.79 (1.21-6.43)	0.02
Hyperlipidemia	0.47 (0.056-3.88)	0.48
Hypothyroidism	0.30 (0.029-3.02)	0.30
Asthma	0.93 (0.33-3.37)	0.93
Body mass index, kg/m^2^	0.99 (0.95-1.03)	0.55
Office SBP, per 10 mmHg	0.99 (0.97-1.01)	0.52
Office DBP, per 10 mmHg	0.99 (0.96-1.03)	0.73
Plasma potassium, mmol/l	1.09 (0.44-2.73)	0.86
Plasma sodium, mmol/l	1.11 (0.93-1.31)	0.25
Plasma creatinine, mmol/l	1.03 (1.01-1.04)	0.002

*SBP* systolic blood pressure, *DBP* diastolic blood pressure, *OR* odds ratio, *CI* confidence interval.

## Discussion

In the present study, we observed a relatively high prevalence of secondary hypertension among under 30-year-old patients undergoing initial hypertension assessment in Finnish tertiary care. Almost half of the patients were diagnosed with secondary hypertension, which is considerably more than what most previous studies including hypertensive individuals of the same age [7–9]. What is more, in this study almost three quarters of the secondary hypertension cases were due to renal disease, whereas non-kidney secondary hypertension was substantially less prevalent. We also observed that the most common renal disease diagnosis was diabetic nephropathy (28.6%). Furthermore, both DM and higher plasma creatinine were associated with increased odds of having secondary hypertension. Given that our study included patients visiting a tertiary care internal medicine clinic for initial hypertension evaluation, these results might not be entirely generalizable to all hypertensive individuals in the community. However, we provide important novel data about the characteristics of secondary hypertension among young adults, which is an understudied topic [7–9]. Our findings could imply that (DM-related) chronic kidney disease account for a significant proportion of secondary hypertension burden among young adults.

Previous studies have reported varying secondary hypertension prevalence rates, while only a few studies have specifically studied this condition among young adults. A previous study from Yoon et al. reported a secondary hypertension prevalence of 16% among hypertensive patients aged 12 to 21 years in the United States [8]. However, most of these patients were treated by primary care physicians, and the prevalence of both primary and secondary hypertension was markedly higher among individuals over 14 years of age. Another study with a study sample of relatively healthy Korean male military personnel aged 19 to 29 years reported a secondary hypertension prevalence of only 2.2% among all hypertensive patients [7]. Both studies had markedly different study settings or study populations compared to our study, which might explain their lower prevalence rates of secondary hypertension. In contrast, a recent study by De Freminville et al. reported a prevalence rate of 29.6% of secondary hypertension among young adults with hypertension, consistent with our findings [9]. The study De Freminville et al. had a somewhat similar study population compared to our study and included hypertensive patients referred to tertiary care for an extensive screening for secondary hypertension. The authors reported secondary hypertension prevalence rates of 27% and 30% among individuals aged 18 to 30 and 18 to 40 years, respectively. However, compared to our study, the main reasons for referral to tertiary care unit included uncontrolled hypertension in primary care, or clinical suspicion of secondary hypertension. Compared to these prior reports, the higher secondary hypertension prevalence observed in our study may be partly attributed to the inclusion of internal medicine clinic patients with pre-existing conditions that may contribute to development of secondary hypertension, such as DM-related renal disease.

Only few prior studies have examined the specific causes for secondary hypertension, specifically among young adults. In this study, we observed fewer patients with endocrine causes for secondary hypertension than what has been described in most previous studies. [9, 17] The study by De Freminville et al. reported primary aldosteronism as the most common cause of secondary hypertension among ≤40 years old individuals, followed by renovascular hypertension, primary kidney disease, and pheochromocytoma or functional paraganglioma. Kim et al. reported kidney disease as the most prevalent cause of secondary hypertension among Korean men aged 19 to 29 years [7]. In that study, renal disease was identified as the underlying cause of secondary hypertension in 68% of individuals. This finding is similar to our study, in which 74% of patients were determined to have secondary hypertension attributable to renal disease. Out of these patients, the most frequent renal disease diagnosis was diabetic nephropathy (28.6% of all patients with renal disease). Furthermore, the high prevalence of DM, and consequently diabetic nephropathy, in our study may partly explain the predominance of renal disease as secondary hypertension etiology. Even though DM is a well-established risk factor for chronic kidney disease in the community [18, 19], given that we included all patients visiting internal medicine clinic for initial examination of hypertension, our study population may be somewhat overrepresented with chronic kidney disease patients compared to the general public. In addition, in patients with chronic kidney disease or DM, distinguishing whether hypertension is the cause or consequence may be challenging due to the presence of coexistent cardiovascular morbidity [20].

Clinical characteristics of young adults with any type of hypertension is an extensively, but inconsistently studied subject [21–27]. These previous studies have reported numerous risk factors, such as obesity, black race, elevated lipids and DM. Instead, limited data exists about the characteristics of young adults with secondary hypertension. The study by Kim et al., which included a rather selected study population reported normal body weight, hematuria, proteinuria, abnormal thyroid function test results and severe hypertension as the most common characteristics predicting secondary hypertension [7]. De Freminville et al. on the other hand found that DM, use of 2 or more antihypertensive medications, and low serum potassium level were associated with having secondary hypertension. [9] Conversely, male sex and family history of hypertension were associated with lower odds of secondary hypertension. In our study, we did not observe association between low potassium level and secondary hypertension, most likely since none of our patients had primary aldosteronism. Yet, we did observe that DM and higher plasma creatinine were associated with secondary hypertension. This finding most likely represents the high observed prevalence of renal parenchymal disease, particularly diabetic nephropathy, among our study population. Nevertheless, since determining whether a patient has DM or an elevated plasma creatinine level through laboratory testing or self-report is both convenient and cost-effective, our findings suggest that screening for secondary hypertension in these patients is a feasible approach also in primary healthcare settings.

Although current American and European hypertension guidelines exhibit some variation, both recommend screening for secondary hypertension primarily based on age (under 30 or 40 years), with possible additional remark regarding hypertension severity. [14, 16] The Finnish national hypertension guidelines similarly recommend to consider screening for secondary hypertension in all hypertensive patients under the age of 30 [28]. However, the guidelines do not provide specific recommendations on how to implement the screening procedure for these individuals in clinical practice. To our knowledge, there is limited or no evidence regarding the cost-effectiveness of large-scale secondary hypertension screening in young adults. In fact, implementing extensive routine screening procedures for all young hypertensive individuals would likely require significant healthcare resources and could potentially result in unnecessary harm and concern for patients. Implementing targeted secondary hypertension screening could therefore reduce the unnecessary burden on public healthcare system while shifting the focus toward early management and treatment of hypertension. Considering that initial kidney function testing and DM diagnosis can be easily carried out with rather inexpensive and straightforward methods, such as a single blood test or a spot urine sample, limiting the use of more extensive diagnostic investigations could be advantageous. Routine screening for secondary hypertension in young adults may be enhanced by initially prioritizing targeted diagnostic evaluations in primary care setting before considering referrals to tertiary care.

The strengths of our study include data collected over a span of 20 years representing real-life clinical patients in Finnish tertiary care. Although our study sample was relatively small, we were still able to include a notable number of young patients with secondary hypertension, considering the rarity of this condition. Our study also has some limitations such as missing data among some included characteristics. As the study population consisted of patients whose hypertension evaluation was performed in tertiary care, the prevalence comorbid conditions, such as DM and chronic kidney disease, was notably high in our sample. What is more, the prevalence of secondary hypertension, particularly due to kidney disease, was higher compared to most previous studies. However, the severity of kidney disease, especially among patients with diabetic nephropathy was relatively mild. Thus, the findings from this study might not be generalizable to all young hypertensive individuals in the community or to patients referred to screening for secondary hypertension due to severe or resistant hypertension.

In this study, we present DM and higher serum creatinine as the most important risk factors for secondary hypertension among young hypertensive tertiary care patients in Finland. Renal parenchymal disease, particularly diabetic nephropathy, accounted for most of the diagnosed secondary hypertension cases. The next most common secondary etiology was obstructive sleep apnea. Other, such as endocrine, causes for secondary hypertension were extremely rare. Our findings suggest that initial secondary hypertension diagnostic strategies might be carried out using rather few diagnostic tests which may may support more restrictive secondary hypertension screening strategies among young hypertensive patients in clinical practice. Additionally, these findings highlight the critical role of primary healthcare in screening and diagnosing secondary hypertension, as individuals with hypertension, DM and kidney disease can be identified through relatively simple diagnostic methods. Although our study population did not only include patients with severe or resistant hypertension, our findings suggest that an extensive diagnostic approach may not be needed for hypertensive adults solely based on young age. However, this study alone does not provide definite evidence on whether young adults should be systematically screened for secondary hypertension. More research is needed to evaluate which young hypertensive individuals would mostly benefit from secondary hypertension screening. Moreover, additional evidence is called for to guide primary diagnostic strategies and referring patients to tertiary care. Nevertheless, because hypertension in young individuals is related to hypertension-mediated organ damage already by midlife and following cardiovascular mortality later in life [29–31], early intervention and treatment strategies remain essential regardless of the underlying etiology. Therefore, minimizing the use of overly complex screening methods for distinguishing between hypertension subtypes may be crucial, as such approaches could delay the initiation of treatment and achievement of optimal BP control in young adults.

## Summary

### What is known about the topic

- There is considerable variation in the previously reported causes and prevalence rates of secondary hypertension among adolescents and young adults, which may be due to differences in study settings and populations.
- Even though hypertension guidelines emphasize the importance of distinguishing between primary and secondary hypertension particularly in young individuals, there is a lack of evidence on whether these patients should be screened for secondary hypertension.

### What this study adds

- Among tertiary care patients under the age of 30, almost half of the patients were diagnosed with secondary hypertension.
- Almost three quarters of the secondary hypertension cases were due to renal disease, whereas other causes of secondary hypertension were rare.
- Although our study population did not only include patients with severe or resistant hypertension, our findings suggest that an extensive diagnostic approach for exclusion of all secondary causes of hypertension may not be needed for all young hypertensive patients.

## Data Availability

The dataset generated and analyzed during the current study is subject to confidentiality laws and is therefore unavailable. The core Finnish legislation protecting this data includes the Act on the Status and Rights of Patients (785/1992), the General Data Protection Regulation (GDPR), and the Act on the Processing of Client Data in Healthcare and Social Welfare (703/2023).
